# Acute Spontaneous Perforation of Rectosigmoid Junction in a Patient with Quadriplegia following Spinal Cord Injury

**DOI:** 10.1155/2020/8881840

**Published:** 2020-09-30

**Authors:** Oshan Basnayake, Chiran Rathnaweera, Umesh Jayarajah, Gishanthan Shanthamoorthy, Heshan Dayantha Siriwardena, Asela Jayathilaka

**Affiliations:** Department of Accident and Orthopedic Services, National Hospital of Sri Lanka, Colombo, Sri Lanka

## Abstract

**Background:**

Patients with cervical spinal injury with quadriplegia are at an increased risk of developing serious gastrointestinal complications. We report an unusual case of spontaneous rectosigmoid perforation in a quadriplegic patient. *Case Presentation*. A 58-year-old man with diabetes mellitus and hypertension presented to the emergency department following a fall from 25 feet of height. He sustained a fifth cervical vertebral fracture with quadriplegia and neurogenic shock. One week later, he developed progressive abdominal distension with tachycardia, low blood pressure, and respiratory distress. His abdomen was soft but had impaired liver dullness. Imaging showed evidence of visceral perforation. He underwent an emergency laparotomy and was found to have a perforation of the antemesenteric border of the rectosigmoid junction with fecal contamination. The perforation was repaired primarily, and a temporary loop ileostomy was created. The patient received intensive care for 4 days, and thereafter, the recovery was uneventful. He was later transferred to the spinal unit for further management. The intraoperative findings, histology, and subsequent colonoscopy did not reveal any underlying cause for the perforation.

**Conclusions:**

Clinical signs and symptoms are generally absent in patients following spinal cord injury, and the diagnosis of serious gastrointestinal pathology can be difficult and challenging. We believe that ischemia at the rectosigmoid junction precipitated by multiple factors was the possible reason for the spontaneous perforation.

## 1. Background

Patients with cervical spine injury or lesions with quadriplegia are at an increased risk of developing serious gastrointestinal complications, and the reported incidence of such complications varies from 4.7 to 6.2% [1, 2]. Only a few sigmoid perforations have been reported in quadriplegic patients, and they all have precipitating factors such as digital evacuation, insertion of enema, and impacted hard stools [3–5]. We report an unusual case of spontaneous perforation of unknown origin in a quadriplegic patient. To our knowledge, this is the first reported case of spontaneous rectosigmoid junction perforation in a patient with quadriplegia.

## 2. Case Presentation

**Figure 1:**
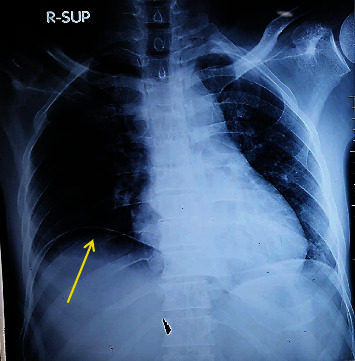
Chest X-ray: yellow arrow shows gas under the right hemidiaphragm.

**Figure 2:**
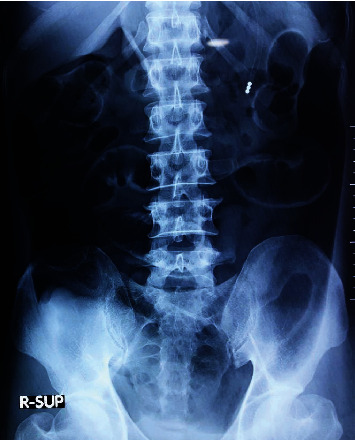
Supine abdominal X-ray shows distended bowel loops with evidence of extramural gas.

**Figure 3:**
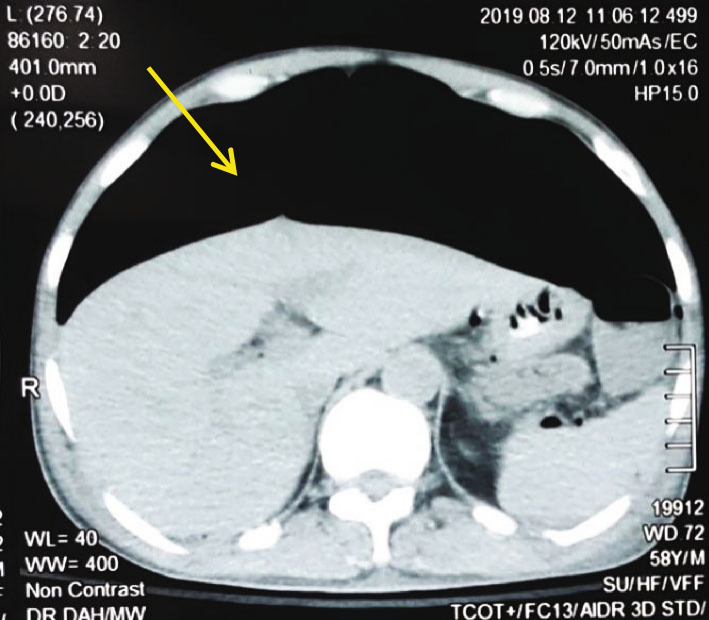
Noncontrast CT scan of the abdomen: yellow arrow shows the presence of intraperitoneal gas.

A 58-year-old man with diabetes mellitus and hypertension for 8 years presented to the accident and emergency department following a fall from 25 feet of height. The initial assessment revealed evidence of complete cervical spinal injury causing quadriplegia (best motor response at C5 level-American Spinal Injury Association (ASIA)-A) and neurogenic shock with a pulse rate of 48 beats per minute and blood pressure of 75/35 mmHg. His respiratory parameters were normal. He did not have any significant head, chest, abdominal, or other injuries. Computed tomography (CT) scan of the cervical spine showed an anterior compression fracture of the fifth cervical vertebral body without significant angulation or retropulsion. His cervical spine was immobilized with a Philadelphia collar. His plain chest X-ray and abdominal ultrasonography were normal on admission. After the initial fluid resuscitation, he was started on noradrenaline infusion via a central venous line for his neurogenic shock. One week later, he developed progressive abdominal distension with tachycardia, low blood pressure, and respiratory distress. On clinical examination, the abdomen was remarkably soft with hyperresonant percussion note and obliteration of the liver dullness. Chest and abdominal radiographs and a noncontrast CT of the abdomen were performed. The chest X-ray showed gas under both hemidiaphragms (Figure 1), and the supine abdominal X-ray showed distended bowel loops with evidence of extramural gas (Figure 2). His noncontrast CT scan confirmed the presence of a significant amount of intraperitoneal gas causing splinting of the diaphragm (Figure 3).

**Figure 4:**
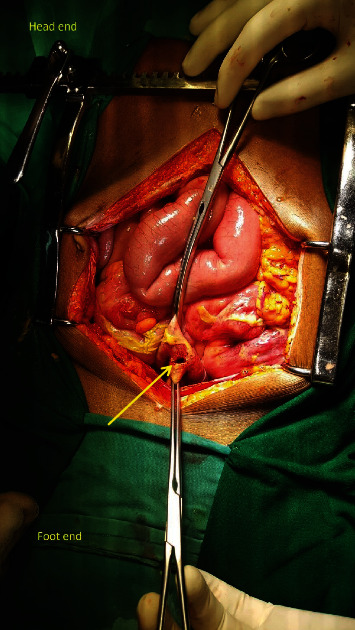
Intraoperative image: yellow arrow shows the perforation of the antemesenteric border of the rectosigmoid junction.

He was aggressively resuscitated and underwent an emergency laparotomy. There was a perforation at the antemesenteric border of the rectosigmoid junction with gross peritoneal contamination with liquid fecal matter (Figure 4). There was no bowel or mesenteric contusion to suggest a blunt impact, and there were no impacted feces in the colon or rectum. Furthermore, the rectum and the sigmoid colon were macroscopically normal and did not suggest an obvious underlying pathology for the perforation. A biopsy was taken from the perforated edge, and a primary repair was performed. A comprehensive peritoneal survey was done which did not reveal any other abnormality. A temporary loop ileostomy was created. The patient received intensive care for 4 days, and thereafter, the recovery was uneventful. His histology report revealed inflammatory infiltrate without any evidence of malignancy.

He was later transferred to the spinal unit for further management. He underwent a colonoscopy later which did not reveal any positive findings.

## 3. Discussion and Conclusions

We present an unusual case of spontaneous bowel perforation in a patient with spinal cord injury and quadriplegia. The atypical and delayed presentation may be explained by the lack of sensory (visceral and somatic) signals and autonomic dysfunction [1]. Therefore, the classical clinical signs and symptoms such as abdominal pain, tenderness, guarding, and rigidity were absent. A study carried out among 132 quadriplegic and 74 paraplegic patients showed statistically significant longer operative time and higher postoperative morbidity and mortality compared with matched controls [2]. This was mainly due to the delayed presentation and significant physiological derangement at the time of surgery [2, 3].

Previously reported cases of intestinal perforations in spinal cord injury patients were mainly described in relation to the stomach, sigmoid colon, and rectum. Gastric perforation was explained by the higher risk of stress ulcers in patients with spinal cord injury [4, 5]. There were only three reported cases of sigmoidal perforation in patients with spinal cord injury [6–8]. Pigac and Masic described a foreign body causing colonic perforation, fecal peritonitis, and death [6]. Rajan et al. described a case of colonic perforation which was precipitated by external suprapubic pressure used for the initiation of micturition (Crede's method) [7]. Stercoral perforation due to faecaloma causing pressure-induced ischemic necrosis of the bowel wall was also reported [8]. Therefore, colonic perforations in spinal cord injury patients were mainly related to stercoral perforation, digital evacuation of feces, and insertion of enemas [9–11].

Our patient had an unusual spontaneous perforation at the rectosigmoid junction. We postulate a few possible explanations for this presentation. Spinal cord injury-induced motility disorder due to failure of external inputs from the autonomic nervous system can cause paralysis and gaseous distention of the lumen. This may precipitate a perforation at an area of preexisting pathology such as neoplasia or diverticular disease. However, in our patient, there was no evidence to suggest an underlying cause for the perforation. There was no bowel or mesenteric contusion to suggest a blunt impact due to trauma, and there were no impacted feces in the colon or rectum to suggest stercoral perforation. Furthermore, the histological analysis of the perforated edge was negative for any neoplastic or inflammatory process. Moreover, his colonoscopy did not reveal any other lesions or diverticula in the colon. However, considering the short duration after trauma, the aetiology of the perforation is more likely to be secondary to the primary injury. A small subserosal tear may have later perforated due to the relative ischemia and the gaseous distension due to the spinal cord injury. Furthermore, an undetected or delayed presentation of the bowel perforation after 1 week of trauma should also be considered.

The perforated segment was related to the Sudeck's critical point which is described as the point of origin of the last sigmoidal artery from the inferior mesenteric artery [12]. This point is relatively avascular and considered as a watershed line. During rectosigmoid anastomosis, this region is thoughtfully avoided to prevent possible ischemia at the anastomotic site [12]. Spontaneous perforation in our patient may be secondary to ischemia at this Sudeck's critical point due to the combination of neurogenic shock and peripheral vasoconstriction of end arteries caused by noradrenaline. Usually, the marginal artery of Drummond acts as the portal of anastomosis between various branches of colonic vessels [13]. However, in this patient, the atherosclerotic process secondary to longstanding diabetes and hypertension may have compromised the blood supply via the margin artery, further aggravating the ischemia at the rectosigmoid region. Furthermore, the perforation occurred at the antemesenteric border which further supports the argument of ischemia-induced perforation. However, the fact that the proximal bowel and mesentery were healthy without any signs of injury is against the argument of ischemia-induced perforation.

An intraoperative decision was made to perform a loop ileostomy as a mode of proximal fecal diversion as there was a risk of poor wound healing and subsequent wound dehiscence.

Clinical signs and symptoms are generally absent in patients following spinal cord injury, and the diagnosis of serious gastrointestinal pathology can be difficult and challenging. A high index of suspicion and early detection can prevent the delay in the management of these patients to reduce morbidity and mortality.

We described an unusual case of spontaneous rectosigmoid perforation in a patient with spinal cord injury and quadriplegia. Clinical signs and symptoms are generally absent in such patients following spinal cord injury, and the diagnosis of serious gastrointestinal pathology can be difficult and challenging. We believe that ischemia at the rectosigmoid junction precipitated by multiple factors was the possible reason for the perforation.

## Data Availability

All data generated or analyzed during this study are included in this published article.

## Abbreviations

CT: Computed tomography
ASIA: American Spinal Injury Association.
